# cAMP signaling microdomains and their observation by optical methods

**DOI:** 10.3389/fncel.2014.00350

**Published:** 2014-10-28

**Authors:** Davide Calebiro, Isabella Maiellaro

**Affiliations:** ^1^Institute of Pharmacology and Toxicology, University of WürzburgWürzburg, Germany; ^2^Bio-Imaging Center/Rudolf Virchow Center for Experimental Biomedicine, University of WürzburgWürzburg, Germany

**Keywords:** G protein-coupled receptor, cyclic AMP, signaling microdomain, fluorescence resonance energy transfer, neurons

## Abstract

The second messenger cyclic AMP (cAMP) is a major intracellular mediator of many hormones and neurotransmitters and regulates a myriad of cell functions, including synaptic plasticity in neurons. Whereas cAMP can freely diffuse in the cytosol, a growing body of evidence suggests the formation of cAMP gradients and microdomains near the sites of cAMP production, where cAMP signals remain apparently confined. The mechanisms responsible for the formation of such microdomains are subject of intensive investigation. The development of optical methods based on fluorescence resonance energy transfer (FRET), which allow a direct observation of cAMP signaling with high temporal and spatial resolution, is playing a fundamental role in elucidating the nature of such microdomains. Here, we will review the optical methods used for monitoring cAMP and protein kinase A (PKA) signaling in living cells, providing some examples of their application in neurons, and will discuss the major hypotheses on the formation of cAMP/PKA microdomains.

## Introduction

The concept of second messenger made its first appearance nearly 60 years ago after the identification of cyclic AMP (cAMP) as the heat-stable and dialyzable molecule that mediated the intracellular actions of the hormones glucagon and epinephrine in the liver (Rall and Sutherland, 1958). Subsequently, cAMP has been shown to regulate a myriad of cellular processes such as gene expression, proliferation, apoptosis, exocytosis or migration as well as a wide range of physiological functions including immune responses, cardiac contractility and memory formation (Beavo and Brunton, 2002).

The events triggering the production of cAMP have long been believed to begin exclusively at the plasma membrane, where a variety of ligands are capable of binding to and activating several members of the large family of G protein-coupled receptors (GPCRs; Pierce et al., 2002; Lefkowitz, 2013). Subsequently, active GPCRs induce the exchange of GTP for GDP on the α-subunit of the Gs protein, which in turn activates adenylyl cyclases (AC). These enzymes, which exist in different isoforms, convert ATP to cAMP, which ultimately stimulates different effector proteins, namely cyclic-nucleotide-gated (CNG) channels, guanine-nucleotide exchanging proteins activated by cAMP (Epac) and protein kinase A (PKA). A group of phosphodiesterases (PDEs), which degrade cAMP to AMP, are finally responsible for bringing the concentration of cAMP back to the basal level, so that the cell can respond to a new stimulus (Beavo and Brunton, 2002).

For many years after the discovery of cAMP, its intracellular concentration has been thought to increase uniformly after stimulation of a receptor located on the plasma membrane. This view was based on at least two important observations: first, cAMP is a small soluble molecule with a high diffusion coefficient in solution; second, dissimilarly from Ca^2+^ ions, which are buffered by binding proteins in the cytosol, cAMP remains largely free in the cytoplasm. However, this view has been challenged by an increasing number of experimental observations, which provide convincing evidence that cAMP may remain confined in microdomains near the sites of its synthesis, thus leading to the selective activation of a subset of effectors located in their proximity.

First indirect evidence for such compartmentation in cAMP/PKA signaling was provided by Corbin et al. (1977) working on heart lysates. Few years later, it was reported that prostaglandin E1 (PGE_1_) and the β-adrenergic agonist isoproterenol caused similar elevations of cAMP in heart tissue, but only isoproterenol was able to activate glycogen phosphorylase (Brunton et al., 1979, 1981; Hayes et al., 1979, 1980). Interestingly, it was subsequently found that PGE_1_ activated PKA only in a soluble fraction, whereas isoproterenol could also activate PKA in a particulate fraction, which was apparently required for its effects on glycogen phosphorylase (Buxton and Brunton, 1983). A number of analogous observations of hormones inducing dissimilar effects in the same tissue have been reported by several groups (Steinberg and Brunton, 2001; Zaccolo, 2011; Lefkimmiatis and Zaccolo, 2014). Moreover, Jurevicius and Fischmeister performed an elegant experiment in which they used a set-up that allowed perfusing the two halves of a frog cardiomyocyte with two different solutions and at the same time record L-type calcium currents induced by β-adrenergic stimulation. Based on their results, they concluded that β-adrenergic receptors (β-AR) induced local cAMP elevations, which caused the preferential activation of nearby Ca^2+^ channels (Jurevicius and Fischmeister, 1996).

However, it was only later, thanks to the introduction of optical methods for monitoring cAMP/PKA signaling in living cells, that a direct observation and investigation of cAMP microdomains became possible. This review will focus on the currently available tools for monitoring cAMP levels and PKA activation in living cells, providing some illustrative examples of their application in neurons. Moreover, we will discuss the major hypotheses on the mechanisms responsible for the formation of such microdomains, which have emerged from these studies.

## Fluorescence resonance energy transfer (FRET) sensor for monitoring cAMP signaling in living cells

The currently available optical methods for monitoring cAMP signaling in living cells are largely based on fluorescence resonance energy transfer (FRET; Förster, 1948). FRET is a physical phenomenon that occurs between two fluorophores located in close proximity: if the distance between two fluorophores is less than approximately 10 nm and there is a certain overlap between their fluorescence spectra, the energy in the excited state of the first fluorophore (donor) can be transferred without emission of a photon to the second fluorophore (acceptor), which will in turn emit a photon of a lower energy. The resulting red-shift of the emitted light, or, alternatively, the shortening of the time the donor resides in the excited state can be used to monitor FRET. Such measurements can be performed in living cells with the spatial and temporal resolution of fluorescence microscopy. This approach has been exploited to follow all the major steps of GPCR signaling, including the generation of cAMP and the activation of PKA (Adams et al., 1991; Zaccolo et al., 2000; Zhang et al., 2001; DiPilato et al., 2004; Nikolaev et al., 2004; Ponsioen et al., 2004)—for a comprehensive review on sensors for monitoring other steps in GPCR signaling see Lohse et al. (2008) and Lohse et al. (2012). Here we will focus on the sensors used for monitoring cAMP concentrations and PKA activation.

The first FRET sensor used to monitor cAMP was based on PKA. This sensor consisted of two PKA catalytic (C) subunits labeled with fluorescein as FRET donor and two PKA regulatory (R) subunits labeled with rhodamine as FRET acceptor—hence the name FICRhR (fluorescein-labeled catalytic subunit and rhodamine-labeled regulatory subunit, pronounced “flicker”). In the presence of low cAMP concentrations, the R and C subunits are associated and FRET can occur between the two nearby fluorophores. When cAMP concentrations increase, cAMP binds to the R subunits causing dissociation of the C subunits and a concomitant loss of FRET. This sensor was used to monitor cAMP signaling in different neuronal preparations, such as invertebrate neurons (Bacskai et al., 1993; Hempel et al., 1996), *Xenopus* embryonic spinal neurons (Gorbunova and Spitzer, 2002) or murine thalamic neurons (Goaillard and Vincent, 2002), as well as in oocytes (Webb et al., 2002; Takeda et al., 2006) and frog ventricular myocytes (Goaillard et al., 2001). A drawback of this sensor was that it had to be injected into the cytoplasm, which was a challenging task, particularly for cells of small size. Moreover, it has been suggested that the injection pipette might work as a “cAMP sink”, artificially reducing the cAMP concentration inside the cell (Vincent and Brusciano, 2001).

These problems were later overcome by Manuela Zaccolo and Tullio Pozzan with the introduction of a genetically-encoded PKA-based sensor (Zaccolo et al., 2000). In this sensor, the PKA R and C subunits were fused with two color variants of GFP—BFP and GFP, later replaced by CFP and YFP, respectively (Zaccolo and Pozzan, 2002). This sensor could now be introduced into cells by co-transfection of two plasmids, each coding for one of the two fluorescently labeled PKA subunit. Although this represented a major advancement, some limitations and possible drawbacks remained. Most importantly, this sensor retained PKA catalytic activity, which can alter intracellular signaling and various cellular functions. Moreover, both labeled subunits had to be overexpressed and present in comparable amounts in order to associate into a functional sensor, which complicated the use of this sensor and increased the chances that it might interfere with the functions of the cell (Rich and Karpen, 2002; Haugh, 2012; Saucerman et al., 2013; Rich et al., 2014).

In an attempt to circumvent the remaining limitations of PKA-based sensors, the group of Martin Lohse in Würzburg developed a new type of cAMP sensor that was single-chain and catalytically inactive. The first version of this sensor was based on a cAMP-binding domain derived from Epac1, a GTP exchange factor for Rap1 activated by cAMP, which was fused on either side to CFP and YFP (Nikolaev et al., 2004)—binding of cAMP to this sensor causes a decrease of FRET between CFP and YFP. In parallel, two other groups generated similar types of sensors that were based on full-length or partially truncated version of Epac proteins (DiPilato et al., 2004; Ponsioen et al., 2004; Dunn et al., 2006). A whole range of single-chain cAMP sensors with improved characteristics have been developed by utilizing cAMP binding domains derived from other proteins, deleting functional domains of Epac proteins, introducing point mutations in the cAMP binding domains and/or using improved donor:acceptor pairs (Nikolaev et al., 2005, 2006; Violin et al., 2006, 2008; Norris et al., 2009; Klarenbeek et al., 2011; Polito et al., 2013).

In addition, endogenous PKA signaling can be monitored by FRET sensors known as AKARs, standing for A-kinase activity reporters (Zhang et al., 2001). This family of biosensors has been generated by sandwiching a PKA substrate sequence and a phosphoamino acid binding domain between a FRET donor (e.g., CFP) and a FRET acceptor (e.g., YFP or Venus). Once phosphorylated by PKA, the PKA substrate contained in the sensor interacts with the phosphoamino acid binding domain, leading to an increase in FRET between the two fluorophores. Like for cAMP sensors, several generations of AKAR sensors with progressively improved characteristics have been produced by replacing the initial fluorophores with brighter and more photostable ones (Zhang et al., 2005; Allen and Zhang, 2006; Depry et al., 2011; Erard et al., 2013; Chen et al., 2014) or introducing a long flexible linker between the PKA substrate and the phosphoamino acid binding domain (Komatsu et al., 2011). It should be noted that, since the phosphorylation of AKAR sensors and the accompanying FRET changes are rapidly reversed by the action of endogenous phosphatases, these sensors measure the equilibrium between phosphorylation and dephosphorylation and not just PKA activity.

Although FRET-based methods for monitoring cAMP/PKA signaling offer several advantages compared to other approaches, there are still some limitations and potential pitfalls that deserve careful consideration—for a detailed review see Rich and Karpen (2002), Haugh (2012) and Rich et al. (2014). One is the already mentioned catalytic activity of some sensors. In addition, all sensors have the potential of interacting with other proteins present in the cell. This risk should be particularly low with the use of small sensors that contain no specific interacting domains. Finally, all sensors have the potential of buffering intracellular cAMP. Performing measurements at different levels of expression of the sensor (Castro et al., 2013) appears to be a good strategy for evaluating the presence of such an unwanted effect.

## Visualization of cAMP/PKA microdomains by FRET microscopy

One of the first applications of the new method was to study cAMP signaling in the giant sensory neurons of the see snail *Aplysia californica*, a model organism that had been extensively used by the group of Eric Kandel to demonstrate the importance of cAMP in synaptic plasticity (Kandel, 2009; Kandel et al., 2014). In this model, serotonin released by interneurons activates a Gs-coupled receptor located on pre-synaptic termini, where it induces both short-term and long-term facilitation. Since the latter requires phosphorylation of CREB as well as mRNA and protein synthesis, it was postulated that the cAMP signals produced in the periphery must reach the soma in order to induce long-term changes in synaptic transmission. Using *Aplysia* sensory neurons injected with FlCRhR, Bacskai et al. (1993) found that bath stimulation with saturating serotonin concentrations induced PKA activation in the distal processes but only a minimal PKA activation near the nucleus. Interestingly, such cAMP gradient between the periphery and the central compartment was observed also with forskolin stimulation and was not prevented by treatment with IBMX, a PDE inhibitor. At the same time, injected cAMP was found to be freely diffusible, with a diffusion coefficient of 780 µm^2^/s. Based on these observations, the authors hypothesized that the observed gradient between the periphery and the soma was due to the higher surface-to-volume ratio of the fine processes compared to the cell body. However, it has been suggested that buffering by FICRhR might artificially favor the formation of cAMP gradients, an effect that could be bypassed if the concentration of injected cAMP should exceed the buffering capacity of the sensor (Rich et al., 2001). This factor may complicate the interpretation of the results of Bacskai et al. (Rich et al., 2001).

Subsequent experiments performed in neurons of the lobster stomatogastric ganglion injected with the same PKA sensor revealed that electric stimulation of afferent fibers, presumably through release of endogenous modulators, is capable of inducing local increases of cAMP in fine neurite branches (Hempel et al., 1996). Interestingly, after repeated electric stimulation for 2–3 min, a unidirectional propagation of cAMP signals from the fine neurites towards the soma was observed. The authors explained this as the intracellular diffusion of cAMP from its peripheral site of production towards the central compartment (Hempel et al., 1996). However, measurements of the time required for the axonal propagation of the cAMP signal gave values that are consistently lower than it would be expected for the free diffusion of cAMP and about 6–8 times lower than those experimentally observed after local cAMP microinjection (Bacskai et al., 1993). Moreover, the results obtained with lobster stomatogastric ganglion neurons were inconsistent with the simple hypothesis that differences in the surface-to-volume ratio might be the major cause for the formation of cAMP gradients between fine peripheral structures and the soma.

Subsequently, optical measurements of cAMP levels and PKA activation in neurons have been performed in various mammalian preparations (Nikolaev et al., 2004; Gervasi et al., 2007; Dunn and Feller, 2008; Neves et al., 2008; Mironov et al., 2009; Castro et al., 2010, 2013; Shelly et al., 2010; Hu et al., 2011; Nicol et al., 2011). Some of these studies provided further evidence for the existence of cAMP/PKA microdomains. For instance, Nikolaev et al. (2004) followed the formation of transient cAMP gradients in hippocampal neurons transfected with the Epac1-camps sensor and locally stimulated with the β-adrenergic agonist isoproterenol. They measured a very rapid diffusion of cAMP inside neurons (diffusion coefficient ~500 µm^2^/s), which was ~20-fold slower if measured with a PKA-based sensor (Nikolaev et al., 2004). These data confirmed the high diffusibility of cAMP in neurons.

In an elegant study, Neves et al. (2008) performed a series of spatial simulations of the propagation of cAMP signals in hippocampal CA1 neurons, which they subsequently validated in rat hippocampal slices transfected with the Epac1-camps sensor. This study confirmed the possible generation of cAMP gradients between fine processes and the cell body as a consequence of different surface-to-volume ratios, despite a homogenous distribution of receptors and AC on the entire plasma membrane (Neves et al., 2008). In agreement with these findings, a study by Castro et al. (2010) in pyramidal cortical neurons imaged by two-photon microscopy revealed higher cAMP and PKA responses to isoproterenol stimulation in small distal dendrites compared to the bulk cytosol.

Another emerging model to study the spatiotemporal dynamics of cAMP/PKA signaling in neurons is represented by *Drosophila melanogaster*, in which several genetic studies have revealed the importance of cAMP/PKA signaling for memory formation and synaptic plasticity (Kandel, 2009). Interestingly, a number of studies have employed FRET sensors to directly monitor cAMP/PKA signaling in semi-intact larval and adult preparations as well as in intact flies (Shafer et al., 2008; Tomchik and Davis, 2009; Gervasi et al., 2010; Duvall and Taghert, 2012; Boto et al., 2014). For example, flies expressing the Epac1-camps sensor have been used to investigate cAMP signaling in the pacemaker neurons of the central nervous system that are involved in circadian rhythms (Shafer et al., 2008; Duvall and Taghert, 2012). These studies provide evidence that the response to the neuropeptide Pigment Dispersing Factor (PDF) in the pacemaker M cells requires the presence of AC3 and the A-kinase anchoring protein (AKAP) Nervy. Interestingly, signaling by other GPCRs in M cells or by the PDF receptor in other pacemaker cells was not affected by depletion of either AC3 or Nervy. These findings suggest a high degree of compartmentation of PDF signaling in M cells.

Another interesting line of research in *Drosophila* focuses on the involvement of cAMP/PKA signaling in olfactory memory, which is processed in specialized brain regions called Mushroom Bodies (MBs). In an interesting study, Tomchik and Davis (2009) used a FRET sensor to monitor cAMP signaling in two regions of the MBs called α-lobe and calix. They found that the responses to dopamine and octopamine differed substantially in the two regions, with the calyx being particularly sensitive to octopamine while dopamine inducing the largest response in the α-lobe. In a subsequent study, Gervasi et al. (2010) analyzed PKA signaling in the different regions of MBs using an AKAR sensor. They suggested that, whereas octopamine can induce a generalized PKA response in MBs, dopamine induces a localized PKA activation in the α-lobe. Interestingly, the selective localization of PKA signals in α-lobes in response to dopamine was lost in mutant flies that carried an inactive Dunce PDE (Gervasi et al., 2010). These studies are consistent with the hypothesis that cAMP/PKA signals might be spatially restricted in MB neurons.

Furthermore, targeting of cAMP or PKA sensors to different signaling complexes by fusion with one of their components, such as type-I and type-II PKA regulatory subunits (Di Benedetto et al., 2008), PDEs (Herget et al., 2008) and ACs (Wachten et al., 2010), has provided additional evidence for the existence of cAMP microdomains in different types of non-neuronal cells. The application of similar approaches to neurons might allow to further investigate the organization of cAMP microdomains in these cells. For instance, Herbst et al. (2011) used an AKAR sensor targeted to the plasma membrane via a K-ras domain to show the existence of a distinct sub-membrane compartment for cAMP signaling in PC12 cells.

## Mechanisms responsible for signal compartmentation

This topic has been thoroughly discussed elsewhere (Willoughby and Cooper, 2007; Conti et al., 2014; Lefkimmiatis and Zaccolo, 2014; Saucerman et al., 2013; Rich et al., 2014). Here we will highlight some general aspects and provide a few selected examples.

**Figure 1:**
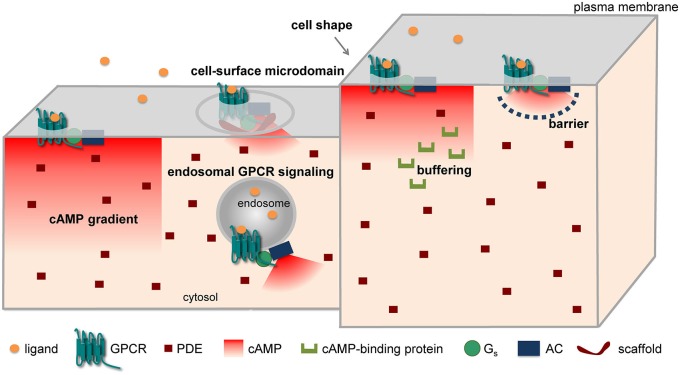
**Major mechanisms implicated in cAMP compartmentation**.

Different mechanisms have been put forward to explain the compartmentation of cAMP/PKA signaling in spite of the high diffusibility of cAMP in solution. These include the formation of cAMP gradients due to reaction-diffusion mechanisms, a different subcellular localization of signaling molecules (receptors, ACs, PDEs, etc.), factors (e.g., fiscal barriers, buffering, higher viscosity) capable of hindering the diffusion of cAMP, and the association of signaling molecules into supramolecular complexes, often through the intervention of scaffolding proteins (Figure 1). Indeed, all these mechanisms could cause local variations in cAMP concentrations, ultimately leading to activation of PKA and downstream targets in a spatially restricted manner.

The formation of cAMP gradients from the plasma membrane to the cell interior has been predicted on the basis of the spatial segregation of ACs on the plasma membrane and PDEs in the cytosol long before the advent of optical methods (Fell, 1980; Kholodenko, 2006; Kholodenko and Kolch, 2008). In addition, differences in the surface-to-volume ratio among different parts of a cell, for instance between the fine neurites and the soma of a neuron, have been claimed for similar reasons to generate a gradient, with cAMP being higher in the finer structures (Kholodenko and Kolch, 2008; Neves et al., 2008). Such gradients have been directly visualized by FRET microscopy in cardiomyocytes (Zaccolo and Pozzan, 2002; Nikolaev et al., 2006, 2010) and in neurons (Bacskai et al., 1993; Neves et al., 2008; Castro et al., 2010; Gervasi et al., 2010).

Another mechanism that has been advocated for the formation of cAMP microdomains involves the non-uniform localization of the proteins implicated in cAMP/PKA signaling in different subcellular structures and/or microdomains. For instance, in cardiomyocytes β_1_-adrenergic receptors (β_1_-ARs) have been suggested to be localized in both caveolar and non-caveolar membrane fractions, whereas β_2_-ARs appear to be prevalently localized in caveolae and to exit caveolae upon activation (Rybin et al., 2000). Interestingly, pharmacological disruption of caveolae enhances and prolongs β_2_-AR-mediated cAMP accumulation (DiPilato and Zhang, 2009). Moreover, combining FRET and scanning ion conductance microscopy, it was shown that functional β_2_-ARs are localized selectively in transverse tubules of cardiomyocytes, whereas β_1_-ARs are distributed across the entire cell surface (Nikolaev et al., 2010). Such selective localization on the surface of cardiomyoctes might explain why stimulation of β_1_- and β_2_-ARs produce different effects although both receptors activate the cAMP signaling pathway. More recently, we and another group have independently found that GPCRs can not only activate ACs once located at the cell surface, but can continue stimulating cAMP production after internalization to an endosomal compartment that contains G-proteins and ACs (Calebiro et al., 2009, 2010; Ferrandon et al., 2009). Very recently, such a phenomenon has also been described for the β_2_-AR (Irannejad et al., 2013). Interestingly, the authors of this study were able to directly visualize active β_2_-ARs and Gα_s_-subunits on endosomal membranes by taking advantage of fluorescently labeled nanobodies that bind to these proteins only when they are in an active state (Irannejad et al., 2013). Similarly, there is increasing evidence for a functional role of GPCRs located on the nuclear membrane (for a review see Tadevosyan et al., 2012). These findings provide a new basis to explain compartmentation in GPCR signaling, and could allow reconciling the notion of spatially confined cAMP/PKA signaling with the known phosphorylation by PKA of targets located deep inside the cell or distant from the site of receptor activation, as might occur in neurons.

A hindered diffusion of cAMP has been advocated by Rich et al. (2000) to explain the results of whole-cell patch clamp experiments in which cAMP concentrations near the plasma membrane were measured using CNG channels. Since they found a low exchange of cAMP between the cytoplasm immediately below the plasma membrane and the bulk cytosol, they hypothesized the presence of a 3D barrier, possibly involving the endoplasmic reticulum, which would limit diffusion of cAMP between these two compartments.

Finally, a number of studies have revealed that different components of GPCR signaling cascades can assemble into functional molecular complexes, thus allowing the formation of signaling domains of the size of a few nanometers (for a detailed review see Lefkimmiatis and Zaccolo, 2014). An illustrative example of such concept is represented by the reported formation of a signaling complex involving the β_2_-AR, heterotrimeric G-proteins, AC, PKA and its target, the L-type Ca^2+^ channel Ca_V_ 1.2 (Davare et al., 2001). Such compartmentalization could explain electrophysiological data obtained in frog ventricular cardiomyocytes that support the preferential functional coupling of β-ARs to nearby L-type Ca^2+^ channels (Jurevicius and Fischmeister, 1996). An important role in this context is played by AKAPs. The over 50 AKAPs identified so far are capable of localizing PKA to multiple subcellular structures via interaction with a specific domain localized at the N-terminus of PKA regulatory subunits. Interestingly, AKAPs do not only interact with both PKA-I and II isoforms, but can bind several other proteins involved in GPCR signaling, such as receptors, ACs, PDEs and phosphatases (Esseltine and Scott, 2013). Thus, these scaffolding proteins appear to play a fundamental role in assembling focal cAMP/PKA signaling complexes. An elegant demonstration of this concept comes from a study by Di Benedetto et al. (2008) in cardiomyocytes. In this study, a FRET sensor for cAMP was fused to either RI- or RII-derived AKAP-binding domains, thus allowing its targeted localization to different compartments. Interestingly, it was found that isoproterenol stimulation causes a larger cAMP elevation at the level of the RII-tethered sensor. In contrast, the RI-tethered sensor was preferentially responding to PGE_1_. Moreover, the two PKA pools had a different sensitivity to different isoform-selective PDE inhibitors and were coupled to different downstream targets (Di Benedetto et al., 2008). This study provided a possible molecular explanation for the observations made by Buxton and Brunton (1983) nearly 25 years before.

Although all these studies have provided fundamental insights into the possible mechanisms of cAMP compartmentation, putting all the pieces of the puzzle together is not trivial. Moreover, some hypotheses on the formation of cAMP gradients are difficult to verify, due to the lack of adequate tools. Here, an important help may come from mathematical models and simulations capable of predicting the behavior of signaling cascades (Kholodenko, 2006; Kholodenko and Kolch, 2008) and, thus, of verifying, in a systematic way, the possible contribution of different mechanisms to cAMP compartmentation. Indeed, several groups have built a series of mathematical models of GPCR/cAMP signaling pathways and performed different types of simulations (e.g., single/multiple compartment vs. spatial, or deterministic vs. stochastic), a topic that has been recently reviewed by Saucerman et al. (2013).

Most of these simulations confirm the importance of having differentially localized AC and PDE activities for the generation of cAMP gradients. Some simulations have suggested that the presence of ACs on the plasma membrane and a higher PDE activity in the cytosol is sufficient to produce a cAMP gradient in the cell. For instance, Oliveira et al. (2010) performed a stochastic simulation of cAMP production and degradation in HEK293 cells, which they validated with previous cAMP FRET measurements. They concluded that the formation of cAMP gradients requires a pool of PDE4D anchored in the cytosol as well as PDE4D phosphorylation by PKA. In a subsequent paper, the same group simulated dopamine D1 signaling in a segment of a spiny projection neuron dendrite, and evaluated the effect of anchoring the receptor/AC and/or PKA in the spine heads *vs*. the dendrite. The results indicated that when receptors and ACs were located on the spine heads, cAMP was significantly higher there than in the dendrite. In addition, PKA activation was predicted to be more efficient when PKA was located together with AC in the same compartment (Oliveira et al., 2012). However, it has been pointed out that these simulations utilized rather high AC and PDE activities, which may not reflect the physiological ones (Saucerman et al., 2013; Conti et al., 2014). On the other hand, simulations by other groups suggest that the formation of a cAMP gradient requires the presence of additional factors capable of reducing the effective cAMP diffusion. Proposed factors include the presence of physical barriers, buffering of cAMP and local changes in the viscosity of the cytoplasm (Saucerman et al., 2013). For instance, Feinstein et al. (2012) applied a mathematical model to study the contribution of all the major mechanisms proposed for the formation of cAMP microdomains in pulmonary microvascular endothelia cells. They concluded that, with experimentally measured PDE and AC activities in these cells, the formation of a gradient could be observed only using a diffusion coefficient for cAMP of 3 µm^2^/s or less, i.e., at least 100-fold lower than experimentally measured in the bulk cytoplasm. However, it should be mentioned that, while mathematical simulations are a very powerful tool for interpreting experimental results and formulating new hypotheses, they also have a number of limitations. First, they are based on simplified models of cellular biochemistry. Second, they also have spatial and temporal limits. In particular, since the size of signaling complexes is in the range of tens to hundreds of nanometers, it would be important to be able to predict the behavior of signaling molecules at such short distances. Third, the results of simulations are largely dependent on the kinetics parameters and initial concentrations used. Unfortunately, for several of these parameters we possess only rough estimations, which are mostly based on *in vitro* measurements, and therefore do not necessarily reflect the values in intact cells. Further efforts to better estimate these parameters would help improving the reliability and predictive power of such simulations.

## Conclusions and future perspectives

The development of optical methods that allow monitoring cAMP/PKA signaling in living cells played a fundamental role in revealing an unexpected level of organization in GPCR signaling cascades. On the one hand, receptors and downstream signaling molecules appear to be highly organized in micro- or nano-domains on biological membranes. Contrary to what originally thought, such high degree of compartmentation appears to also involve a rapidly diffusing molecule such as cAMP. On the other hand, the finding of receptor signaling on endosomes and possibly other intracellular compartments adds another level of organization, which could help explaining why receptors coupled to the same downstream signaling pathway can induce dissimilar biological effects. It is likely that new technological developments, as is often the case, will play a fundamental role in further elucidating the organization of signaling microdomains located on the cell surface and on other intracellular membranes. Indeed, optical methods with higher temporal and spatial resolution would permit to better dissect the nature of such microdomains. In this context, methods based on single molecule microscopy, which have been recently applied by us and other groups to GPCRs (Hern et al., 2010; Kasai et al., 2011; Calebiro et al., 2013), appear particularly indicated to analyze the composition of protein complexes on the surface of living cells and to capture dynamic events such as protein-protein interactions with high temporal and spatial resolution. Furthermore, new models such as *Drosophila* and new methods such as two-photon microscopy are hopefully going to permit a direct observation of these phenomena *in vivo*, thus allowing to address the role of cAMP compartmentation under highly physiological conditions. After all, almost six decades after the discovery of cAMP, there are still many good reasons to expect novel and exciting discoveries in this field.

## Conflict of interest statement

The authors declare that the research was conducted in the absence of any commercial or financial relationships that could be construed as a potential conflict of interest.
